# Evidence-based practice for individuals or groups: let’s make a difference

**DOI:** 10.1007/s40037-013-0071-2

**Published:** 2013-07-31

**Authors:** M. de Groot, J. M. van der Wouden, E. A. van Hell, M. B. Nieweg

**Affiliations:** 1 Professorship in Health Care and Nursing, Hanze University of Applied Sciences, P.O. Box 3109, 9701 DC Groningen, the Netherlands; 2 University Medical Centre Groningen, University of Groningen, Antonius Deusinglaan 1, 9713 AV Groningen, the Netherlands; 3 School of Nursing, Hanze University of Applied Sciences, P.O. Box 3109, 9701 DC Groningen, the Netherlands

**Keywords:** Evidence-based practice, Research utilisation, Delivery of health care, Health education, Professional competence

## Abstract

The aim of applying science into practice is to deliver high-quality health care. Thinking about teaching the necessary accompanying skills, a distinction can be made between using evidence for individual patient care and using scientific knowledge for the development of protocols or guidelines for groups of patients or professionals. In this paper, these two ways of applying science into practice are being considered. We plea for explicating the differences between the individual patient and a group of patients or professionals when applying scientific knowledge in the decision-making process. The acknowledgment of these differences facilitates the teaching of the accompanying competences and different CanMEDS roles.

## Introduction

Evidence-based practice (EBP) is essential in assuring quality of health care. The individual patient is an explicit part in the definition of EBP [1]. However, the term EBP is not restricted to decision-making processes for individual patients only, but also used for the development of guidelines and procedures for groups of patients or caregivers [2]. Obviously, applying evidence to answer a clinical question that was raised for an individual patient differs from the use of scientific proof to justify the care for patient groups or guideline development for professional behaviour. EBP for a single patient is often the responsibility of a single caregiver, whereas EBP for groups is the responsibility of a team of health care providers [3]. The lack of differentiation between these two approaches leads to fuzziness when it comes to which competences are needed in education and practice. We therefore advocate to be more explicit and aim to clarify the distinction between EBP for the individual patient and for a group of patients or caregivers by discussing the following five steps: ask, acquire, appraise, apply and assess [4]. Furthermore, we discuss the impact of this differentiation on education.

## Individual patients versus groups of patients or caregivers

### Step 1: ask a searchable question

Critical appraisal of literature starts with formulating a question. For a single patient, the question is typically raised during interaction between patient and caregiver. The question is about an individual patient’s problem and motivated by the need to adapt care as usual to individual preferences and responses. Acquiring this type of question is typically associated with the CanMEDS competency of communicator. For groups, the question arises as a consequence of a recurring problem in the clinic or may be raised during conference, in interaction with colleagues or while reading the latest, scientific journals (CanMEDS scholar). Here, the question is motivated by the need to improve the quality and consistency of care by a group of caregivers. So, the origin of a question for a single patient differs from a question for groups.

### Step 2: acquire information

The strategy to search best evidence differs. For a single patient, one uses keywords that fit the patient’s characteristics specifically. Due to time constraints, quick actions are often needed and all sources of evidence are considered. For groups, keywords that fit the average patient are used and only the highest level of evidence is selected. This process may well be performed by more than one researcher (CanMEDS collaborator). As a consequence, finding evidence is often time-consuming. Acquiring evidence for a single patient needs a narrow search strategy; acquiring evidence for groups of patients or professionals requires a broad search strategy.

### Step 3: appraise search results

The critical appraisal of the evidence is part of the CanMEDS competency of scholar. Appraisal may differ between a single patient and groups. If a high level of evidence is unavailable, other sources are handbooks, case studies or an expert’s opinion. In many cases a randomized clinical trial or systematic review does not completely reflect the individual patient’s circumstances. Decision-making for groups requires research of high scientific rigor. Appraisal takes place using an explicit, systematic process. An innovation is not ready for application in practice if an appropriate and valid research base (proof) is unavailable. To answer a question for a single patient, research is considered a source of evidence; to answer a question for a group of patients or caregivers, research is the only source of evidence [5].

### Step 4: apply the evidence in practice

The implementation of evidence into clinical practice for single patients and groups differs fundamentally. The decision-making for a single patient is in many cases done by a sole professional, using implicit and personal methods [6]. The impact of the implementation of EBP is on the level of an individual patient and as such documented in the patient file. The decision-making for groups is a process performed by a team of professionals and characterized by discussions and consensus. For efficacious implementation, the outcome typically involves the development of protocols or guidelines, which requires all clinicians (on the level of a department, a hospital or even (inter)national) to change their behaviour and to stick to the new guideline or protocol. This requires change in the routine management, which renders implementation a time-consuming process often guided by a special team of several (organizational) experts [7]. Affinity with the CanMEDS competencies of manager and collaborator is needed to implement a new guideline or protocol. As a consequence this process may well take several years. Applying evidence for a single patient is about personalizing health care; applying evidence for a group is about optimal quality and consistent health care or professional behaviour.

### Step 5: assess the provided care

Finally, the evaluation processes differ. For a single patient, a caregiver provides the necessary monitoring and evaluates the decision with the patient (CanMEDS communicator). For groups, a systematic evaluation is needed to assess its true value for a population and professionals’ adherence (CanMEDS collaborator, manager and scholar). Evaluating decisions for a single patient is a practitioner’s routine; evaluating decisions for a group of patients or caregivers is a scientist’s routine.

## EBP and scientific education

**Fig. 1:**
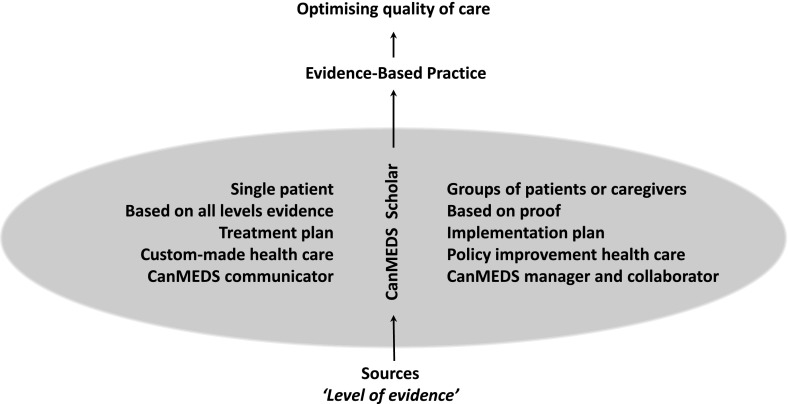
Schematic overview of the differences in evidence-based practice for a single patient as opposed to a group of patients or caregivers

EBP is about bringing scientific results into practice. In itself EBP does not discriminate between the decision-making processes for an individual patient or groups of patients or professionals. In the above-mentioned paragraphs we have explained where and how the critical appraisal, the decision-making process and the implementation differs. These differences are also expressed in the accompanying caregivers’ competencies. For example, a health care expert as defined by the CanMEDS Framework must integrate all competencies in daily practice in order to provide optimal, ethical and patient-centred health care. Decision-making for an individual patient is an integrative part of daily practice, but requires the CanMEDS competencies of communicator and scholar specifically. Decision-making for groups is about the optimization of care in general. Since it deals with group issues it is also about the development of the organization and the profession in general. It requires management and innovation. The decision-making process for a group is therefore more related to the CanMEDS competencies of manager, collaborator and scholar. During our EBP teaching in baccalaureate, Master’s and postgraduate programmes, we have experienced that the distinction between decision-making for a single patient or a group helps students and professionals to better understand what competences need to be developed to apply science into practice (Fig. 1).

## Research utilization as component of EBP

We are not the first to reflect on the difference between single patients or groups. For the latter, alternative terminology can be found in the literature: research utilization (RU), knowledge valorization (KV), evidence-based quality management and several others [3]. RU is to our knowledge the oldest and most authentic terminology that describes the use of research findings for groups [8]. We support the views of Levin and Feltman [5] who regard EBP as a broader concept than RU. We therefore suggest the use of RU as a term to describe the decision-making process for a group which results in development and implementation of protocols or guidelines. EBP can be regarded as the umbrella that encompasses both agency and individual activities which aim to use research evidence in practice [9].

It can be argued that RU and EBP should be taught together, because the methodology, *i.e.* the process of critical appraisal, is similar. Also, one can reason that discrimination between *dealing with* science and *contributing to* science is far more important than the division between RU and EBP. Nevertheless, we think that the distinction between EBP and RU is an important one to make, especially in educational programmes. The differences and similarities in competences needed for EBP and RU can give insight into a student’s qualities and preferences for a future career. This also helps students to understand the rationale for evidence-based guidelines, which greatly improves their acceptance and use in clinical practice, especially when the evidence contradicts current practice [3]. The distinction between EBP and RU also helps curriculum developers to establish a valid scientific framework throughout the educational programme.

## Conclusion

Inspiring students to use scientific knowledge throughout their career will make them ‘lifelong learners’ and fosters an inquisitive spirit. So, if we really want to improve practice, all health care students need to be confronted with the use of science in every lecture and in every assignment. One cannot be a professional and accountable practitioner without using and applying scientific knowledge. The distinction between single patients and groups of patients or professionals helps curriculum developers to establish a valid framework throughout the educational programme. We suggest the use of RU as a term to describe the decision-making process for a group of patients or professionals which results in the development and implementation of protocols or guidelines. EBP can be regarded as the umbrella that encompasses both agency and individual activities which aim to bridge the gap between research and practice.

## Essentials

- A distinction can be made between using evidence for individual patient care and using scientific knowledge for the development of protocols or guidelines for groups of patients or professionals.
- Different competences are necessary when applying evidence to answer a clinical question that was raised for an individual patient as opposed to the use of scientific proof to justify the decisions for groups.
- Using evidence to optimize care for the individual patient is associated with the CanMEDS competencies of communicator and scholar.
- The use of scientific proof to justify the care for groups is associated with the CanMEDS competencies of manager, collaborator and scholar.
- Our plea is that educators pay attention to the above-mentioned differences in order to achieve a better understanding by students on how to optimize health care. To distinguish between single patients and groups helps students and professionals to clarify what knowledge and skills are necessary to provide excellent health care.
